# Lack of SOCS3 increases LPS-induced murine acute lung injury through modulation of Ly6C(+) macrophages

**DOI:** 10.1186/s12931-017-0707-6

**Published:** 2017-12-29

**Authors:** Zhilong Jiang, Zhihong Chen, Liyang Li, Wenjun Zhou, Lei Zhu

**Affiliations:** Department of Pulmonary Medicine, Zhongshan Hospital, Fudan University, 180 Feng Lin Road, Shanghai, 200032 China

**Keywords:** Acute lung injury, Ly6C(+) macrophages, SOCS3, JAK/STAT3 signaling

## Abstract

**Background:**

SOCS3 (suppressor of cytokine signaling 3) is a negative regulator of JAK/STAT3 signaling pathway and participates in the regulation of lung inflammation in a mouse model with acute lung injury (ALI). However, it is not well understood how SOCS3 regulates lung inflammation in the ALI mouse model.

**Method:**

In the present study, we investigated the effects of SOCS3 on modulation of Ly6C(+) monocyte phenotypes in a mouse model with lipopolysaccharide (LPS)-induced ALI. Conditional SOCS3(Lyz2cre) mice with myeloid cell-restricted depletion of SOCS3 gene were created by breeding transgenic Lyz2Cre mice with SOCS3(fl/fl) mice. Wilde-type (WT) and SOCS3(Lyz2cre) mice were intratracheal instilled with 5 mg/kg LPS for 2 days. Lung, bronchoalveolar lavage (BAL) and blood were collected for analysis by flow cytometry, ELISA, qRT-PCR and Western blot analysis.

**Results:**

The studies in the ALI mouse model revealed that myeloid cell-restricted SOCS3 deficiency exacerbated the severity of ALI as compared to the WT mice. The increased severity of ALI in SOCS3-deficient mice was associated with higher populations of neutrophils, T lymphocytes and Ly6C(+) monocytes in the inflamed lung tissues. In addition, CCR2 and CXCL15 were elevated, and accompanied by greater expression and activation of STAT3 in the lung of SOCS3-deficient mice. SOCS3-deficient bone marrow-derived macrophages (BMDMs) expressed a higher amount of TNF-alpha, and adoptive transfer of the SOCS3-deficient Ly6C(+) BMDMs into WT mice enhanced the severity of ALI than adoptive transfer of WT control BMDMs. However, depletion of Ly6C(+) circulating monocytes by anti-Ly6C(+) neutralizing antibody moderately attenuated neutrophil infiltration and resulted in lower prevalence of Ly6C(+) cells in the lung of treated mice.

**Conclusion:**

Myeloid cell-restricted lack of SOCS3 induced more severe ALI through modulation of Ly6C(+) subtype macrophages. The results provide insight into a new role of SOCS3 in modulation of Ly6C(+) monocyte phenotypes and provide a novel therapeutic strategy for ALI by molecular intervention of macrophages subtypes.

## Background

Acute respiratory distress syndrome (ARDS) is characterized by endothelial damage, edema and uncontrolled lung inflammation. Corticosteroid is commonly used for the treatment of ARDS, but the therapeutics do not improve patient survival rate [1, 2]. To seek an effective therapeutic approach to ARDS, it is important to study the pathological mechanisms of ARDS in the ARDS animal model. However, currently there is no ideal animal model that mimicks the human ARDS. Mice with acute lung injury (ALI) are commonly used as the animal model, which is established through intratracheal (i.t.) administration with high doses of lipopolysaccharide (LPS), reminiscence to, but not the completely same as human ARDS, because LPS-treated animals undergo lung inflammation resolution and fibrosis 7 days after LPS treatment [3], the clinical feature does not occur in human ARDS. Combinational i.t. instillation of LPS with hydrochloric acid (HCl) or oleic acid (OA) into animals may cause more severe ALI, but evidence of long-term and uncontrolled pulmonary inflammation are not well defined so far [4–6]. Currently the underlying mechanisms of uncontrollable lung inflammation in ARDS patients are still elusive. It is documented that lung alveolar macrophages (AMs) and circulating monocytes are involved in the pathogenesis of human ARDS as well as ALI in the animal model. These cell components play pivotal roles in the development of acute lung injury and uncontrollable lung inflammation. Our previous study in LPS-induced ALI mouse model revealed that depletion of AMs resulted in more severe ALI, where blockade of circulating monocyte migration attenuated ALI. The results revealed the pro-inflammatory function of circulating monocytes [7]. Modulation of circulating monocyte function is suggested as a potential therapeutic approach in the treatment of ARDS/ALI. It is known that suppressor of cytokine signaling 3 (SOCS3) significantly affects macrophage biological function by suppressing the Janus kinase (JAK)/Signal transducer and activator of transcription 3 (STAT3) signaling pathway. Modulation of SOCS3 expression has a significant impact on the process of many macrophage-involved diseases [8–10].

It was previously reported that SOCS3 gene expression was up-regulated in ALI [7, 8, 11]. The increased SOCS3 may help to avoid production of excessive pro-inflammatory cytokines-induced tissue damage in vivo by suppressing JAK/STAT3 signaling pathway. In addition, a recent study showed that activated macrophages can release a soluble form of SOCS3 protein, that is then up-taken by epithelial cells [12]. The role of SOCS3 in ALI has been recently studied in animal models. It was reported that lack of SOCS3 induced more severe ALI in LysMCre-SOCS3fl/fl mice, with more activation of macrophages and elevated Th1/Th17 cell differentiation [10, 11, 13]. Thereby, SOCS3 has an immune regulatory role in the pathogenesis of ALI.

However, recently the immune suppressive function of SOCS3 was challenged by results from other animal models, in which SOCS3 may have pro-inflammatory function. For example, a study in human monocytes showed that knock-down of SOCS3 expression by short interfering RNA induced suppression of some pro-inflammatory cytokine expression, but increases in the expression of anti-inflammatory M2 macrophage markers [14]. Therefore, SOCS3 has properties of both immune regulatory and pro-inflammatory immune functions in different animal models. The underlying molecular mechanisms are still unclear.

Though the effects of SOCS3 on ALI were previously studied in LysMCre-SOCS3fl/fl mice, the underlying mechanisms were not fully investigated [13]. It is known that lymphocyte antigen 6 complex-positive Ly6C(+) and Ly6C(−) monocytes have different immunological functions [15, 16]. Ly6C(+) monocytes are mainly produced from bone marrow progenitors, and can be activated and recruited into the inflamed sites after exposure to pathogens. It was reported that depletion of Ly6C(+) macrophages reduced house dust mite-induced allergic lung inflammation and expression of IL-13 [17]. Therefore, Ly6C(+) monocytes are considered pathogenic during disease development [18]. However, Ly6C(−) macrophages are considered as tissue-resident sentinels, that can release pro-inflammatory cytokines/chemokines and attract circulating neutrophils and Ly6C(+) macrophages towards inflammatory sites [19, 20]. CX3CR1 and CCR2 are considered important for Ly6C(−) and Ly6C(+) monocyte trafficking, macrophage polarization, and pulmonary vascular remodeling [21]. There are increased both inflammatory Ly6C(+) and resident Ly6C(−) monocyte subsets in blood and lung tissues of mice with hypoxia-induced pulmonary hypertension (PH) [21]. CX3CL1/CX3CR1 signaling is considered important in differentiation of Ly6C(−) macrophages and induction of tissue fibrosis in a mouse model with unilateral ureteral obstruction [15], because lack of CX3CR1 expression induced renal pro-inflammatory macrophage proliferation and kidney fibrosis through increasing expression of profibrotic mediator TGF-beta [22]. Therefore, Ly6C(+) and Ly6C(−) macrophages play an important role in the progression of inflammatory diseases. However it is unknown whether the cell phenotypes have impact on ALI and whether SOCS3 expression affects differentiation of the Ly6C(+) and Ly6C(−) macrophage phenotypes. To address these issues, in this study we investigated the effects of SOCS3 on Ly6C(+) monocyte differentiation as well as the role of Ly6C(+) cells in the severity of ALI. Our results revealed that myeloid cell-restricted SOCS3 deficiency exaggerated murine acute lung injury, accompanied with more Ly6C(+) macrophages. However, depletion of Ly6C(+) circulating monocytes attenuated ALI severity. The results have important implications for the development of novel therapeutics in ARDS through molecular intervention of macrophage phenotypes.

## Methods

### Establishment and identification of myeloid cell-restricted SOCS3 knock-out mice

**Fig. 1:**
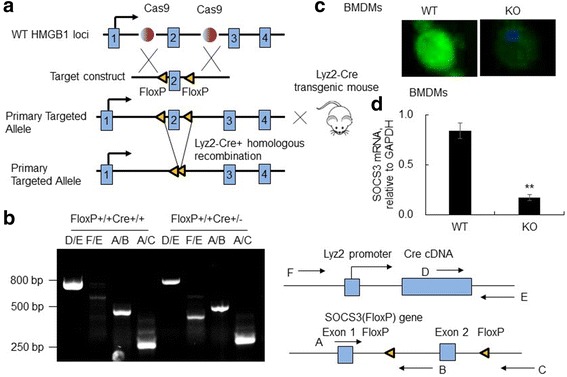
Generation and identification of SOCS3(Lyz2cre) mice. **a** Schematic diagram for generation of SOCS3(Lyz2cre) mice. Floxp-flanked SOCS3 mouse was back-crossed with Lyz2-Cre transgenic mouse to create myeloid cells-restricted SOCS3 knock-out mouse at SOCS3 genome exon 2. **b** The SOCS3(Lyz2cre) mice were identified by PCR analysis. The Cre + and Cre- loci were identified as 700 by (D/E) and 350 bp (F/E) respectively. The FloxP-flanked exon 2 and exon 2-deleted null SOCS3 loci was identified as 420 bp (A/B) and 250 bp (A/C). **c** Identification of SOCS3 protein expression in bone marrow-derived macrophages (BMDMs) derived from WT and SOCS3(Lyz2cre) mice. Intracellular SOCS3 protein was stained with rabbit anti-SOCS3 antibody (Green). One representative photograph was shown. **d** SOCS3 mRNA transcripts in BMDMs were analyzed by qRT-PCR analysis. Data was presented as mean ΔΔCt relative to GAPDH ± standard error. ** *p* < 0.01 compared to WT BMDMs, *n* = 4

The FloxP-flanking SOCS3 transgenic (SOCS3fl/fl) mice were created by Nanjing Biomedical Research Institute of Nanjing University, Jiangsu, China and bred in the animal facility at the Zhongshan Hospital of Fudan University (Shanghai, China). SOCS3 conditional SOCS3(Lyz2cre) mice were generated by serial breeding of SOCS3fl/fl mice with Lyz2-Cre transgenic mice under the control of myeloid cell-restricted lysozyme 2 (Lyz2) promoter (Fig. 1a). Exon 2 was deleted by Cre protein in SOCS3 Floxp+/+/Lyz2Cre+/− or SOCS3 Floxp+/+/Lyz2Cre+/+ mice. Deletion of SOCS3 in SOCS3(Lyz2cre) mice was identified by PCR methods using 4 pairs of primers (Table 1) that analyzed the existence of FloxP and Cre loci respectively. The Cre + and Cre- loci were identified as 700 by (D/E) and 350 bp (F/E) respectively. The FloxP-flanking exon 2 and exon 2-deleted null SOCS3 loci were identified as 420 bp (A/B) and 250 bp (A/C) (Fig. 1b). The mice identified as FloxP+/+Cre+/+ or FloxP+/+Cre+/− phenotypes were considered SOCS3(Lyz2cre) mice with myeloid cell-specific deletion of SOCS3 gene.

**Table 1:** Primers used for detection of gene expression

Gene	Sense (5′-3′)	Antisense (5′-3′)
mSOCS3	GATTTCGCTTCGGGACTAG	CGGCGGCGGGAAACTTGCTG
mIL-1beta	AGAGCTTCAGGCAGGCAGTA	AGGTGCTCATGTCCTCATCC
mIL-6	CAACGATGATGCACTTGC	GTACTCCAGGTAGCTATG
mMCP-1	CCAGCAAGATGATCCCAATG	TTCTTGGGGTCAGCACAGAC
mCXCL15	TGGATCCTGATGCTCCATGG	AGAGGCTTTTCATGCTCAAC
mGAPDH	TGTTCCTACCCCCAATGTGT	TGTGAGGGAGATGCTCAGTG
Floxp-A/B	CGGGCAGGGGAAGAGACTGT	GGAGCCAGCGTGGATCTGC
Floxp-A/C	CGGGCAGGGGAAGAGACTGT	AGTCCGCTTGTCAAAGGTATTGTCCCAC
Cre-D/E	CCCAGAAATGCCAGATTACG	CTTGGGCTGCCAGAATTTCTC
Cre-F/E	TTACAGTCGGCCAGGCTGAC	CTTGGGCTGCCAGAATTTCTC

To identify SOCS3 expression in macrophages, bone marrow cells were flushed from the femurs and tibiae of WT or SOCS3(Lyz2cre) mice. These cells were cultured in RPMI 1640 medium containing 10% FBS and 20 ng/ml of murine M-CSF (PeproTech, Rockhill, NJ) for 7 days to obtain bone marrow-derived macrophages (BMDMs). Cell purity was determined by FACS analysis for CD11b and F4/80 (>96%). SOCS3 protein was stained with rabbit anti-SOCS3 antibody (ImmunoWay Biotech Co., Plano, TX) for 2 h (1:500 dilution) and followed by staining with FITC-conjugated secondary antibody. Cell nuclei were stained with DAPI (Fig. 1c). In addition, SOCS3 mRNA transcripts in SOCS3(Lyz2cre) and WT mice were analyzed by qRT-PCR analysis using primers spanning exon 2 and exon 3 (Table 1). Glyceraldehyde 3-phosphate dehydrogenase (GAPDH) mRNA transcripts were used as internal controls. The data are presented as mean ± standard error, relative to internal control GAPDH (Fig. 1d).

### Animal procedure

8–10 week old male wild-type C57BL/6 mice were obtained from the Shanghai Biomodel Organism Science & Technology. Animal protocol was reviewed and approved by the laboratory animal care and use committee of Zhongshan Hospital, Fudan University. WT and KO mice were i.t. instilled with 5 mg LPS /kg (Sigma- Aldrich, St Louis, MO) as groups WT/LPS and KO/LPS under anesthesia with intraperitoneal (i.p.) administration of 50 mg/kg pentobarbital. The WT and KO mice were i.t. instilled with PBS as controls (Groups WT/PBS and KO/PBS). 2 days after the treatment, lung tissues and bronchoalveolar lavage (BAL) were collected for analysis. Cells in BAL were counted manually. The protein in BAL was measured by BCA kit (Shanghai Beyotime Biotech). Lung tissue was fixed in 4% paraformaldehyde (PFA) and stained with Hematoxylin and Eosin (H&E) for histopathological examination. The lung histology was viewed under a light photomicroscope and evaluated for pathological changes using a double-blind method. The severity of lung injury was evaluated using a semi-quantitative histological index, including alveolar edema, hemorrhage, alveolar septal thickening, and infiltration of polymorphonuclear leukocytes.

For adoptive transfer of BMDMs into recipient mice, BMDM cells-derived from WT and SOCS3(Lyz2cre) mice were labeled with PKH26 red fluorescence (Sigma, Saint Louis, Missouri). A total of 0.4×10^6^ PKH26-labeled cells were i.p. administered into WT mice, in conjunction with i.t. 5 mg/kg LPS. BAL, lung tissue and blood were collected 2 days after the treatment for analysis.

For depletion of blood circulating Ly6C(+) monocytes in SOCS3(Lyz2cre) mice, 25 μg rat monoclonal anti-mouse neutralizing Ly6C antibody (Clone: 6C3, EMD Millipore Corp., Temecula, CA) was i.p. injected into mice, in combination with i.t. instillation with 5 mg/kg LPS (Group anti-Ly6C/LPS). The mice received the same doses of both normal polyclonal goat IgG (R&D systems Inc., Minneapolis, MN) and LPS (group IgG/LPS, *n* = 5) or received PBS alone (group PBS) were used as controls. Lung tissues and BAL were collected 2 days after the treatment for analysis.

### Flow cytometry

Lung digests were obtained by incubation with 1 mg/ml collagenase A and 100 ng/ml DNase (Sigma, St Louis, MO) for 1 h. 0.5–1 ×10^6^ cells from lung digests and BAL were stained with antibodies including PerCP-cy5.5 conjugated anti-F4/80 (eBioscience), APC-Cy7 conjugated anti-CD11b, PE-Cy7 conjugated anti-Ly6G, FITC-conjugated anti-Thy1.2, FITC-conjugated anti-CCR2 (BD Biosciences), APC-conjugated anti-Ly6C and APC-conjugated anti-CD206 (BioLegend. San Diego, CA). For intracellular staining, BMDMs were pre-treated with or without 100 μM STAT3 inhibitor VII (EMD Millipore Corp., Billerica, MA) for 1 h, then stimulated with 500 ng/ml LPS for 18 h. 1 μg/ml Brefeldin A was added in the last 4 h. After the cells were stained with antibodies against the cell surface protein, the cells were then treated with Fixation/Permeabilization buffer (BD Pharmingen, San Jose, CA), followed by incubation with FITC conjugated anti-TNF-alpha antibody (BD Biosciences, San Diego, CA). Analysis was performed on FACScan cytometer (Becton Dickinson, Mountain View, CA). All data were analyzed on Flow Jo software (Tree Star, San Carlos, CA).

### ELISA assay for cytokines

CXCL15 and IL-17 protein concentration in BAL and lung digests were measured by ELISA kit with strict adherence to the manufacturer’s instructions (R&D systems Inc., Minneapolis, MN).

### Western blot analysis

The expression levels of STAT3 protein in lung digests were analyzed by Western blot analysis. 40 μg cell lysates were separated by 10% sodium dodecyl sulfate-PAGE and transferred to hybond-enhanced chemiluminescence (ECL) nitrocellulose membrane (Amersham Pharmacia Biotech, UK). After incubation with blocking buffer containing 5% skim milk in TBST (12.5 mM Tris-HCl pH 7.5, 68.5 mM NaCl, 0.1% Tween 20) for 1 h, the blots were then incubated with primary antibodies for 2 h, including rabbit anti-total STAT3 (Clone: 79D7), rabbit anti-phosphorylated STAT3 at residue Tyr705 (Cell Signaling Technology, Danvers, MA). The anti-mouse GAPDH antibody was used as a loading control. The blots were washed with TBST buffer and incubated with horseradish peroxidase (HRP)-conjugated anti-rabbit immunoglobulin (Ig) (Amersham Biosciences), and then developed with ECL substrate solution (Amersham Biosciences). After incubation with stripping buffer for 15 min at room temperature, the blots were incubated with rabbit anti-murine GAPDH for detection of internal protein loading control.

### Quantitative RT-PCR (qRT-PCR)

TRIzol reagent (Invitrogen, Grand Island, NY) was used to isolate total RNA from lung tissues and BMDMs. The complementary DNA (cDNA) was synthesized from 1 μg of total RNA with ReverTra Ace qPCR RT Master Mix kit (Toyobo Co., Ltd., Osaka, Japan). qRT-PCR was performed using SYBR green PCR Master Mix-Plus (Toyabo Co. Ltd., Osaka, Japan). All primers were synthesized by Shanghai BioSune Biotechnology. The primer sequences are listed in Table 1. GAPDH gene expression was used as an internal control. Real-time PCR reaction was performed on 7500 real-time PCR systems (AB applied Biosystems) under the following conditions: 94 °C 5 min, 40 cycles (94 °C for 30 s, 57 °C for 30 s, 72 °C for 40 s). The relative gene expression was analyzed using the 2-ΔΔCT method.

### Statistical analysis

Results are presented as mean ± standard error (SE) for each group. A Student’s *t* test was used to determine statistical significance between two groups. One-way analysis of variance (ANOVA) followed by Bonferroni’s post-hoc test was performed for parametric multivariable analysis on IBM SPSS statistics V22.0 software. Mann-Whitney U test was used for statistical analysis of non-parametric value. The data were considered statistically significant for *p* values less than 0.05.

## Results

### Lack of SOCS3 expression in macrophages induced more severe acute lung injury in mice after LPS i.T. Treatment

To investigate the role of SOCS3 in acute lung injury, we created conditional SOCS3 KO mice. The SOCS3(Lyz2cre) mice were created by breeding SOCS3fl/fl mice with transgenic Cre mice under the control of Lyz2 promoter, in which exon 2 of SOCS3 loci was deleted in myeloid cells, such as monocytes, macrophages, neutrophils and dendritic cells (Fig. 1a). To identify SOCS3 deficiency in macrophages-derived from bone marrow (BMDMs). Immunostaining analysis that SOCS3 protein expression was observed in WT mouse-derived BMDMs. In contrast, the SOCS3 protein expression was significantly reduced in BMDMs from SOCS3(Lyz2cre) mice (Fig. 1c). Further quantitative analysis by qRT-PCR showed that SOCS3 mRNA transcripts were significantly suppressed in the BMDMs from SOCS3(Lyz2cre) mice for at least 80% as compared to the WT mice (Fig. 1d, *p*<0.01, *n* = 4).

**Fig. 2:**
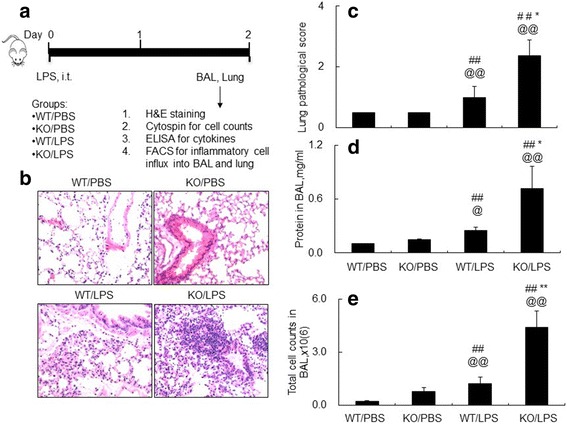
Lack of SOCS3 induced more severe acute lung injury in SOCS3(Lyz2cre) KO mice after LPS treatment. **a** Schematic diagram of WT and KO mice intratracheal (i.t.) treated with 5 mg/kg LPS (WT/LPS and KO/LPS groups). The mice i.t. treated with PBS were used as negative controls (WT/PBS and KO/PBS groups). **b** Lung histology by H&E staining showed exaggerated acute inflammatory infiltration, alveoli destruction, epithelial cell hyperplasia in KO/LPS group than WT/LPS group. **c** Quantitative analysis of acute lung injury by H&E staining. The score of severity was evaluated by scale from 0 to 4 in terms of infiltrated inflammatory cells and alveoli destruction. One representative data of 3 independent experiments was shown. **d** Total protein concentration and (**e**) cell counts in BAL were measured by BCA assay and manually counting. *n* = 6. ## < 0.01 v.s. WT/PBS; * *p* < 0.05, **p < 0.01 v.s. WT/LPS group; @*p* < 0.05, @@*p* < 0.01 v.s. KO/PBS

To investigate whether a lack of SOCS3 in myeloid cells affects acute lung injury in LPS-induced mouse model, WT and KO mice were i.t. treated with 5 mg/kg LPS. The WT and KO mice were i.t. treated with the same volume of PBS as naïve controls. BAL and lung tissues were collected 2 days after the treatment (Fig. 2a). We observed severe acute alveoli destruction, epithelial cell hyperplasia and inflammatory infiltrates in the LPS-treated WT mice (WT/LPS group) compared to the PBS-treated control (WT/PBS group) (Fig. 2b). Furthermore, lack of SOCS3 (KO/LPS group) resulted in acute lung injury that was two times more severe compared to the LPS-treated mice (WT/LPS group) (Fig. 2c) (*p* < 0.05, *n* = 6). Thereby, lack of SOCS3 expression in myeloid cells increased the severity of ALI. In consistent with the results, the total protein content (D) and cell counts (E) in the BAL of SOCS3 KO mice were significantly increased by 3–4 times, compared to those of WT/LPS group (*p* < 0.05, *n* = 6).

### SOCS3 deficiency in macrophages enhanced LPS-induced acute lung inflammation

**Fig. 3:**
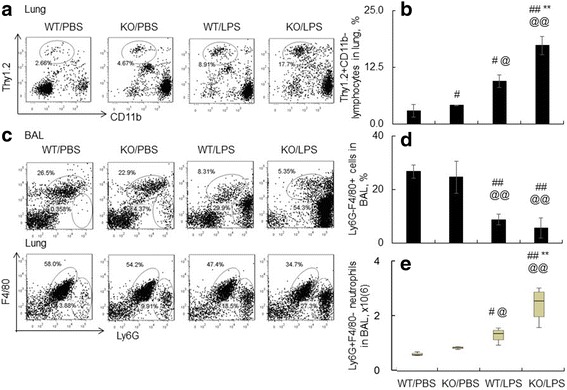
Lack of SOCS3 increased T lymphocytes and neutrophils in the lung of mice after LPS treatment. **a** Representative dot plot data for T cell lymphocytes in the lung tissues of treated mice. **b** T cell lymphocytes were identified as Thy1.2(+)CD11b(−) cells and quantitatively analyzed. **c** Representative dot plot data for macrophages and neutrophils in BAL and lung tissues of the treated mice. Macrophages were identified as Ly6G(−)F4/80(+) cells and neutrophils were identified as Ly6G(+)F4/80(−) cells. **d** The percentage of Ly6G(−)F4/80(+) macrophages in BAL. **e** Neutrophils in BAL was calculated and data was presented as × 10^6^ cells per mouse and data was shown as box and whisker plot. Mann-Whitney U test was used for statistical analysis. #*p* < 0.05, ##*p* < 0.01 v.s. WT/PBS; **p < 0.01 v.s. WT/LPS group, @*p* < 0.05, @@*p* < 0.01 v.s. KO/PBS, *n* = 6

ALI/ARDS is characterized by diffuse and uncontrollable lung inflammation [1]. To define whether SOCS3 deficiency improves acute lung inflammation. We analyzed infiltrating T lymphocytes and neutrophils in 4 groups of mice by flow cytometry. Our results indicated that the population of Thy1.2(+) T lymphocytes in the lung of LPS-treated WT mice were 3 times higher compared to the mice treated with PBS control (Fig. 3a-b). Furthermore, lack of SOCS3 further increased the population of Thy1.2(+) T lymphocytes (KO/LPS group), compared to those in WT/LPS (9.49±1.36 v.s. 17.37±2.00%) (Fig. 3a-b) (*p* < 0.01, *n* = 6). In addition, the percentages of Ly6G(+)F4/80(−) neutrophils in BAL and lung tissues of KO/LPS group were largely increased compared to the WT/LPS control (Fig. 3c), but the percentage of Ly6G(−)F4/80(+) macrophages were relatively decreased (Fig. 3d). In addition, lack of SOCS3 induced 1.8 times more neutrophil cell number in BAL than those in WT/LPS group (Fig. 3e).

**Fig. 4:**
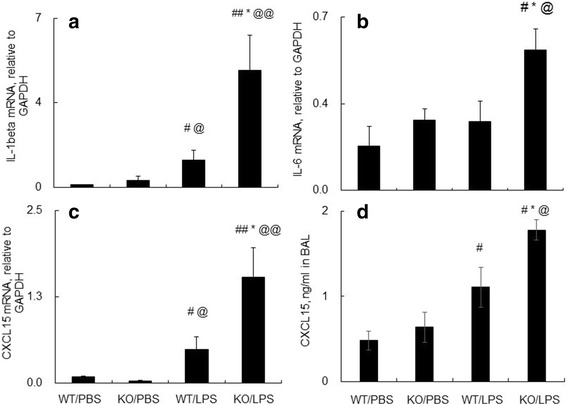
Lack of SOCS3 increased the expression of pro-inflammatory cytokines and chemokines in the lung tissues and BAL of mice after LPS treatment. IL-1beta (**a**), IL-6 (**b**) and CXCL15 (**c**) mRNA transcripts in lung tissues were analyzed by qRT-PCR analysis. Data was presented as ΔΔCT of target genes relative to GAPDH. CXCL15 protein (**d**) in BAL of the treated mice were measured by ELISA analysis. Data were shown as mean value ±standard error. #*p* < 0.05, ##*p* < 0.01 v.s. WT/PBS; * *p* < 0.05 v.s. WT/LPS group, @*p* < 0.05, @@*p* < 0.01 v.s. KO/PBS, *n* = 6

Further analysis by qRT-PCR showed that myeloid cell-restricted deficiency of SOCS3 protein significantly elevated the expression of IL-1beta, IL-6 and CXCL15 mRNA transcripts (Fig. 4a-4c) (*p* < 0.05, *n* = 6) in the lung tissues of LPS-treated KO mice, compared to the LPS-treated WT mice. In addition, more expression of CXCL15 protein was observed in BAL of LPS-treated KO mice than that in LPS-treated WT mice (Fig. 4d). The CXCL15 expression level was correlated with the population of lung CD11b(+)Ly6G(+) neutrophils (data not shown, R^2^ = 0.7427). Similar positive correlation was also observed between IL-17 protein levels and population of lung neutrophils (data not shown, R^2^ = 0.8518).

### Lack of SOCS3 expression increased Ly6C(+) macrophage phenotype in LPS-induced acute lung injury

**Fig. 5:**
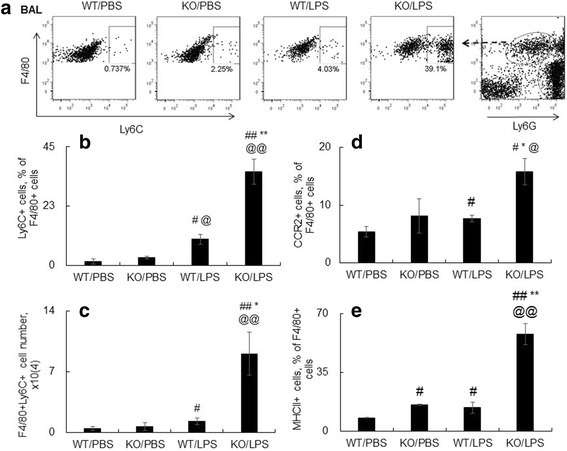
Lack of SOCS3 induced more Ly6C(+) macrophages in BAL of mice after LPS treatment. **a** One representative dot plot data for Ly6C+ macrophages in BAL. Macrophages were gated on F4/80(+)Ly6G(−) cells by flow cytometry. **b** Quantitative analysis of Ly6C(+) subtype macrophages. Data was presented as percentage Ly6C(+) of F4/80+ cells. **c** The number of Ly6C(+) macrophages in BAL were calculated and presented as mean ± standard error ×10^4^. The expression of (**d**) CCR2 and (**e**) MHCII in F4/80(+) macrophages were quantitatively analyzed. #*p* < 0.05, ##*p* < 0.01 v.s. WT/PBS; * *p* < 0.05, **p < 0.01 v.s. WT/LPS group; @*p* < 0.05, @@*p* < 0.01 v.s. KO/PBS, *n* = 5

In the LPS-treated mice we observed a significantly reduced percentage of Ly6G(−)F4/80(+) macrophages in BAL and lung of WT/LPS or KO/LPS group, compared to the PBS-treated control mice (*p* < 0.05) (Fig. 3d), that may be caused by overwhelming influx of neutrophils. However, the total macrophage cell number was increased (data not shown). We further investigated the effects of SOCS3 deficiency on macrophage Ly6C(+) subtypes by flow cytometry analysis. As a result, we observed an increased prevalence of Ly6C(+) macrophage subtype after LPS i.t treatment in WT mice, compared to the PBS-treated WT controls (0.737% v.s. 4.03%). Lack of SOCS3 further increased the Ly6C(+) macrophage population from 2.25% to 39.1% in BAL (Fig. 5a-b). The absolute number was increased from 1.25 ± 0.35 to 9.05 ± 2.47 × 10^4^ cells (*p* < 0.05, *n* = 5, Fig. 5c). The Ly6C(+) cells were positively correlated with neutrophil cell number (R^2^ = 0.7086). Thus, the Ly6C(+) macrophage phenotype was critically involved in the lung inflammation of ALI. Further analysis by flow cytometry also showed higher activation of Ly6C(+) macrophages in KO mice than those in WT mice, as demonstrated by greater expression of CCR2 and MHCII molecules (*p* < 0.05 and *p* < 0.01, *n* = 5, Fig. 5d and e).

**Fig. 6:**
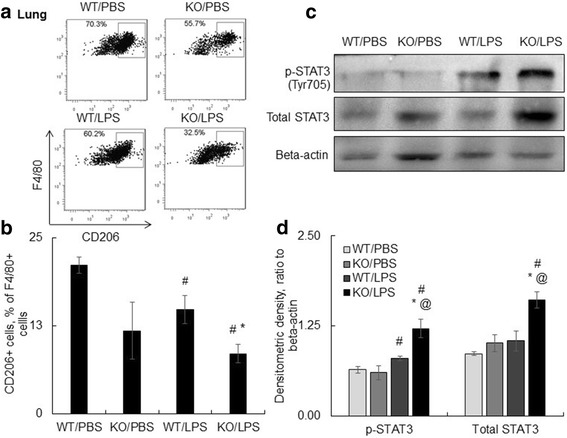
Lack of SOCS3 resulted in lower CD206(+) alternatively activated macrophages (M2 macrophages) and more expression of STAT3 in the lung of mice. **a** CD206+ Macrophages were gated on F4/80(+)Ly6G(−) cells by flow cytometry. One representative dot plot data shown lower CD206+ M2 macrophages in lung digests of KO mice than the WT mice. **b** Quantitatively analysis of M2 macrophages. #*p* < 0.05 v.s. WT/PBS; * *p* < 0.05 v.s. WT/LPS group, *n* = 5. **c** One representative Western blots showed protein levels of total STAT3 and phosphorylated STAT3 at residue Tyr705 in the lung protein extracts. Beta-actin protein was detected as an internal loading control. **d** The protein expression levels were quantitatively analyzed by Image J software and the data was presented as ratio of target protein densitometric density to beta-actin densitometric density. #*p* < 0.05 v.s. WT/PBS; * *p* < 0.05 v.s. WT/LPS group, @*p* < 0.05 v.s. KO/PBS, *n* = 5

Ly6C(+) macrophages are considered pro-inflammatory cell subtypes and may share similar functional properties of classically activated macrophages called M1 cells, whereas Ly6C(−) cells may have anti-inflammatory M2 cell properties which are responsible for inflammation resolution and tissue fibrosis [17, 23, 24]. Further analysis showed that Ly6C(+) macrophages did not express the CD206 protein, and a portion of Ly6C(−) macrophages expressed the CD206 protein, indicating the Ly6C(+) and Ly6C(−) populations have properties of M1 and M2 macrophage phenotypes (data not shown), that results were consistent with previous reports. Our further analysis in SOCS3 KO mice indicated that SOCS3 deficiency moderately reduced CD206(+) M2 macrophages in naïve mice and LPS i.t. treatment further reduced M2 macrophages from 14.81 ± 1.98% to 8.54 ± 1.26% in the lung digests (*p* < 0.06, *n* = 5, Fig. 6a and b). The M2 cell population was negatively correlated with total cell counts in BAL (data not shown, R2 = 0.8658). Therefore, lack of SOCS3 increased Ly6C(+) macrophages, but reduced M2 macrophages. Further investigation by Western blot analysis showed more expression and activation of total STAT3 in KO/LPS group than WT/LPS group ((*p* < 0.05, *n* = 5, Fig. 6c-d).

### Lack of SOCS3 expression in BMDMs increased Ly6C(+) macrophage differentiation with more expression of TNF-alpha

**Fig. 7:**
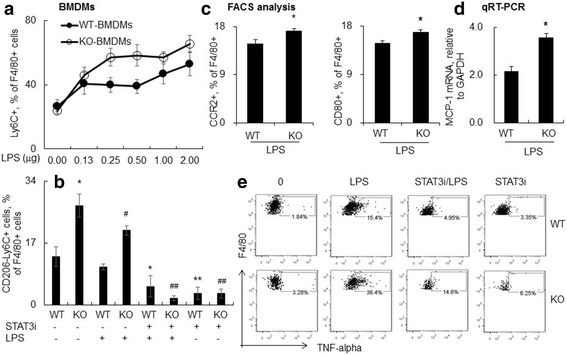
Lack of SOCS3 induced more population and activation of Ly6C+ macrophages in bone marrow-derived macrophages (BMDMs). **a** BMDMs from SOCS3(Lyz2cre) (KO) and wild-type (WT) mice were stimulated with different concentration of LPS for 24 h. Ly6C(+) macrophages were analyzed by flow cytometry analysis. Data was presented as mean ± standard error of Ly6C(+) cells percentage in F4/80(+) macrophages, *n* = 4. **b** Quantitatively analysis of CD206(−)Ly6C(+) macrophages in BMDMs with or without 1 h pre-treatment of 100 μM STAT3 inhibitor (STAT3i). BMDMs cells were stimulated with 500 ng/ml LPS with or without 1 h pre-treatment of STAT3i. 24 h post-LPS stimulation, CD206(−)Ly6C(+) macrophages were analyzed by flow cytometry analysis. Cells were gated on F4/80(+) cells. * *p* < 0.05, **p < 0.01 v.s. WT/0; #*p* < 0.05, ##*p* < 0.01 v.s. KO/0, *n* = 4. **c** Quantitatively analysis of CCR2 (left panel) and CD80 (right panel) expression in BMDMs by FACS analysis. **d** Quantitatively analysis of MCP-1 mRNA transcripts in BMDMs by qRT-PCR analysis. Data was presented as ΔΔCt relative to GAPDH. * *p* < 0.05 v.s. WT/LPS, *n* = 4. **e** TNF-alpha protein expression in CD11b(+)F4/80(+) BMDMs was analyzed by intracellular staining. One representative dot plot data was shown

To further confirm the effects of SOCS3 on Ly6C(+) macrophage differentiation in vitro, we isolated and cultured WT type BMDMs for 7 days. 24 h after stimulation with different concentrations of LPS, we observed the elevated Ly6C(+) macrophages in a LPS concentration-dependent manner (Fig. 7a). However, lack of SOCS3 expression from SOCS3(Lyz2cre) mice-derived BMDMs further increased Ly6C(+) macrophage population, supporting the results in vivo. In addition, blockade of STAT3 activation by pre-treatment of cells with 100 μM STAT3 inhibitor VII significantly resulted in lower Ly6C(+) macrophages under the blockade of STAT3 activation (Fig. 7b). The results further confirmed the regulatory role of SOCS3 through STAT3 signaling in vitro. Additional study by flow cytometry revealed more expression of CCR2 and CD80 in BMDMs lack of SOCS3 in BMDMs (*p* < 0.05, *n* = 4, Fig. 7c). The MCP-1 mRNA transcripts were also more detected by qRT-PCR analysis (*p* < 0.05, *n* = 4, Fig. 7d). The elevated activation of KO BMDMs resulted in 2-fold increases in the expression of TNF-alpha compared to those of WT BMDMs with or without LPS stimulation, and pre-treatment with STAT3 inhibitor VII can reverse TNF-alpha expression-induced by lack of SOCS3 expression (Fig. 7e). Consistent with the results in vivo, the lack of SOCS3 in BMDMs induced differentiation and activation of Ly6C(+) subtype macrophages through JAK/STAT3 signaling pathway.

### Adoptive transfer of SOCS3-deficient BMDMs improved cell migration into the lung of mice with ALI

**Fig. 8:**
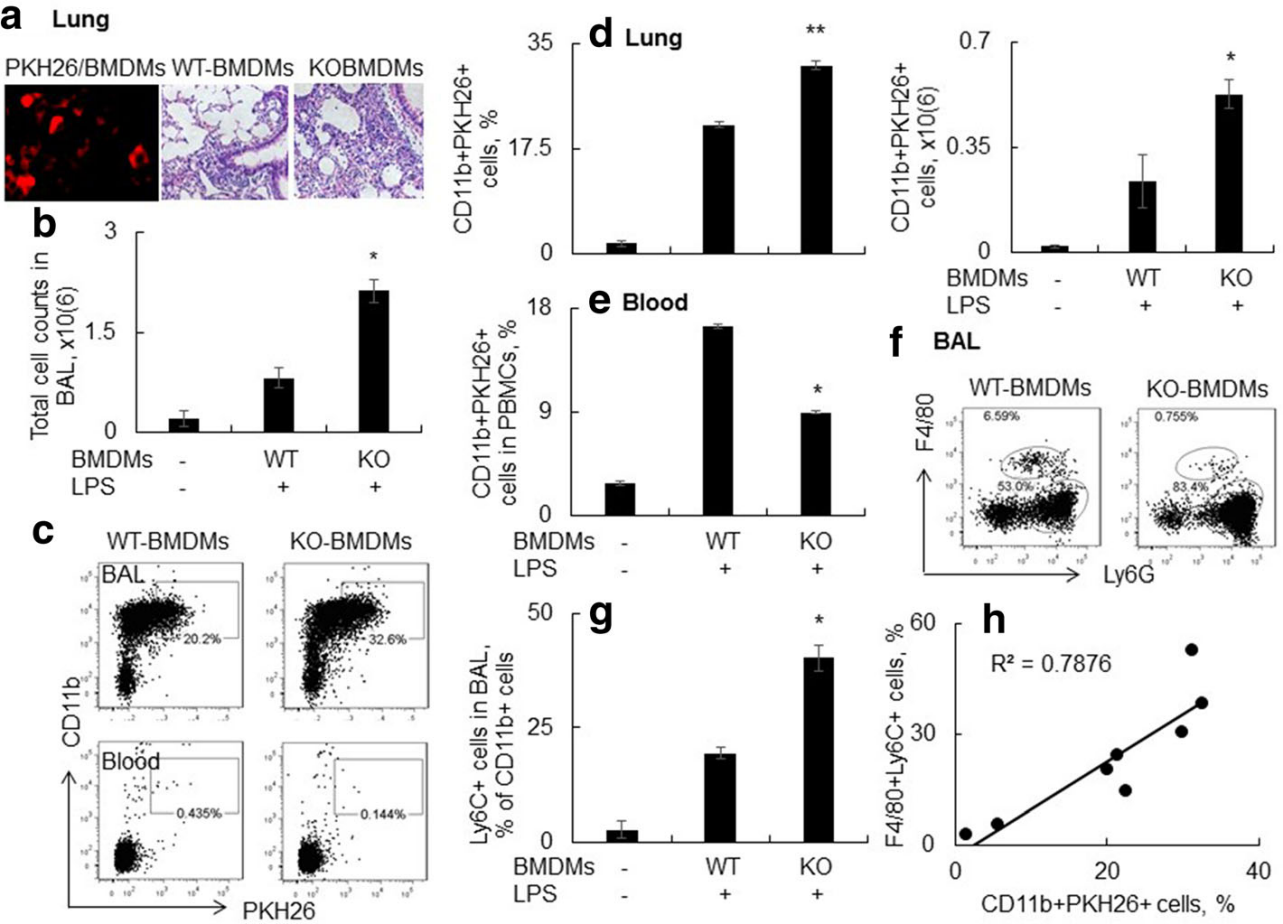
Adoptive transfer of SOCS3-deficient BMDMs into WT recipient mouse induced more severe ALI and BMDMs emigration into the lung. 0.4×10^6^ PKH26-labeled BMDMs from WT and KO mice were intraperitoneal (i.p.) injected into WT mice, in conjunction with i.t. treatment with 5 mg/kg LPS. The mice treated with PBS were used as negative controls. 2 days after cell adoptive transfer, BAL, lung tissue and blood were collected for analysis. **a** PKH26-labeled BMDMs (Left panel) were observed in the lung tissues of KO mice 2 days after adoptive cell transfer. Lung histology of recipient mice was analyzed by H&E staining after adoptive transfer of WT-BMDMs/LPS or KO-BMDMs/LPS. One representative photograph was shown (Right panel). **b** Total cell counts in BAL of groups WT-BMDMs/LPS and KO-BMDMs/LPS. The naive mice were used as controls. * *p* < 0.05 v.s. WT-BMDMs group, *n* = 5. **c** The presence of PKH26-labeled exogeneous BMDMs in lung and blood was analyzed by flow cytometry analysis. Exogenous BMDMs were CD11b(+)/PKH26(+) cells. One representative data was shown. BMDMs in lung (**d**) and blood (**e**) were quantitatively analyzed. * *p* < 0.05, ** *p* < 0.01 v.s. WT-BMDMs group, *n* = 5. **f** Lung neutrophils were analyzed by FACS and F4/80(−)Ly6G(+) cell population was considered as neutrophils. One representative data was shown. Adoptive transfer of SOCS3-deficient BMDMs into WT recipient mouse induced more neutrophil infiltrates into the lungs. **g** Quantitatively analysis of Ly6C(+) macrophage subtypes in BAL. * *p* < 0.05 v.s. WT-BMDMs group, *n* = 5. **h** Correlation analysis between Ly6C(+) subtype macrophages and PKH26-labeled BMDMs in BAL. Each point represents individual sample

Because SOCS3 deficiency increased the expression of MCP-1 (Fig. 7d), CCR2 and MHCII (Fig. 5d and e), we extended our study to further investigate whether more Ly6C(+) macrophage in the lung tissues of SOCS3-deficient mice was caused by greater monocyte migration from blood circulation. To address this issue, we cultured BMDMs from WT and KO mice for 7 days. Then 0.4 × 10^6^ cells were labeled with fluorescent dye PKH26 and adoptively transferred into WT recipient mice in conjunction with LPS i.t. treatment. 2 days after adoptive transfer, we observed the existence of PKH26-labeled BMDMs in the lung tissues (Fig. 8a, left panel), but there was no existence of the cells in the lung tissues of BMDMs-untreated mice (data not shown). Further investigation showed that the recipient mice that received KO-BMDMs developed more severe acute lung injury and inflammation than those that received WT-BMDMs control cells (Fig. 8, right panel). The detrimental effects were accompanied with significantly more cell counts in BAL (Fig. 8b, *n*=5, *p* < 0.05). Flow cytometry analysis showed 2–4 times more PKH26-labeled CD11b(+) BMDMs population in the lungs of mice treated with KO-BMDMs than the mice treated with WT-BMDMs (Fig. 8c, upper panel and 8D). In contrast, the KH26-labeled CD11b(+) BMDMs population was 2 times attenuated in the blood circulation of mice treated with KO-BMDMs than the mice treated with WT-BMDMs (Fig. 8c, lower panel and 8E, *p* < 0.05, *n* = 5). The results indicated that there was more BMDMs emigration from blood circulation into the inflamed lung tissues to exert pro-inflammatory function after adoptive transfer of KO BMDM cells.

The greater emigration of KO BMDMs than WT BMDMs towards lung increased Ly6G(+)F4/80(−) neutrophils (Fig. 8f) and Ly6C(+) subtype macrophages (Fig. 8g) in BAL. In addition, the exogenous BMDMs emigration was positively correlated with the Ly6C(+) subtype macrophages in the inflamed lung (Fig. 8h, R^2^ = 0.7876), indicating the contribution of BMDMs to the increasing population of lung Ly6C(+) macrophages. Therefore, SOCS3 deficiency facilitated acute lung injury and lung inflammation, possibly through increasing migration of pro-inflammatory Ly6C(+) subtype macrophages from blood circulation into the inflamed lung tissues.

### Depletion of Ly6C(+) monocytes by neutralizing antibody ameliorated acute lung injury and lung inflammation in vivo

**Fig. 9:**
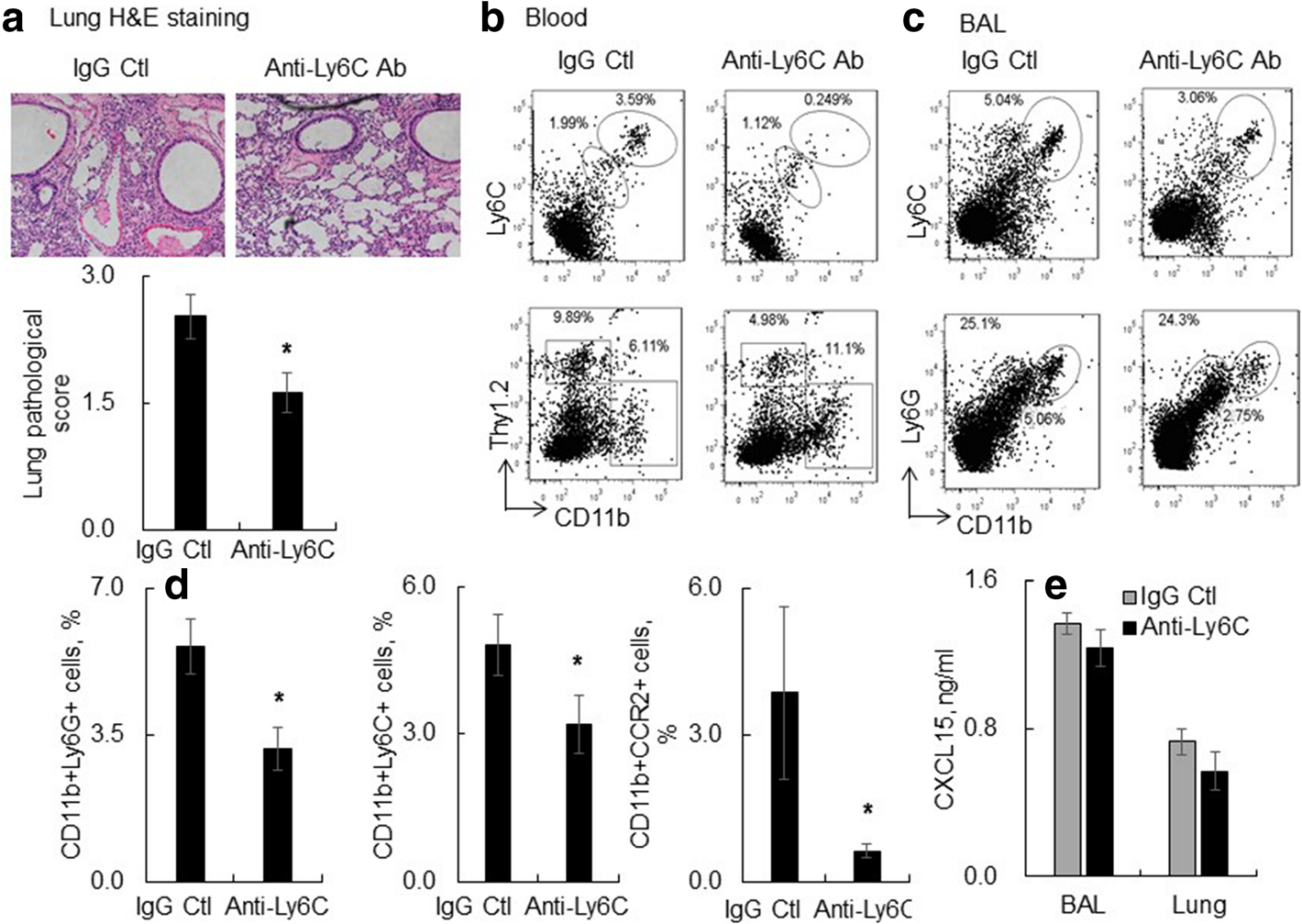
Depletion of circulating Ly6C(+) monocytes suppressed acute lung injury and inflammation in SOCS3(Lyz2cre) (KO) mice after i.t. LPS treatment. 25 μg rat monoclonal anti-mouse Ly6C antibody (Clone: 6C3) and 5 mg/kg LPS were co-administered into mice via i.p. and i.t. respectively (Group anti-Ly6C/LPS, *n* = 5). The mice received the same doses of IgG and LPS were used as controls (group IgG/LPS, *n* = 5). 2 days after the treatment, BAL, blood and lung tissues were collected for flow cytometry analysis. **a** Lung histology analysis by H&E staining. Anti-Ly6C antibody decreased acute lung injury and inflammation. One representative photograph was shown for each group of mice (Upper panel) and quantitatively analysis of the severity of acute lung injury (Lower panel). *p < 0.05 v.s. the IgG control group, *n* = 5. **b** Anti-Ly6C antibody moderately suppressed CD11b(+)Ly6C(+) monocytes and CD11b(−)Thy1.2+ T lymphocytes in blood circulation. **c** Anti-Ly6C antibody moderately suppressed Ly6C(+) macrophages and Ly6G(+) neutrophils in BAL. One representative data of each group was shown. **d** Quantitative analysis of Ly6G(+) neutrophils, Ly6C(+) macrophages and CCR2(+) expression in lung macrophages by flow cytometry. *p < 0.05 v.s. the IgG control group, *n* = 5. **e** CXCL15 protein expression in lung lysates was analyzed by ELISA assay. Data is presented as mean ± standard error

To further investigate the role of Ly6C(+) monocytes in the progression of ALI, we depleted Ly6C(+) monocytes in blood circulation by i.p. instillation of 25 μg rat monoclonal anti-mouse neutralizing Ly6C antibody (Clone: 6C3), in combination with i.t. instillation of 5 mg/kg LPS. As a result, a total of 93% CD11b(+)Ly6C(+) monocytes in blood circulation were depleted by anti-Ly6C antibody treatment. Depletion of Ly6C(+) subtype monocytes in blood circulation significantly reduced the severity of acute lung injury as demonstrated by lower destruction of alveoli and infiltration of inflammatory cells in the lung tissues (Fig. 9a-b, *p*<0.05, *n* = 5). In addition, depletion of circulating Ly6C(+) monocytes reduced approximately 2-fold of circulating Thy1.2(+)CD11b(−) T lymphocytes and Ly6C(+) monocytes (Fig. 9b). Similarly, the Ly6C+ macrophages and Ly6G(+) neutrophils were significantly reduced in BAL of anti-Ly6C antibody-treated mice (Fig. 9c-d). Further analysis by flow cytometry and ELISA indicated that the reduced lung inflammation in the Ly6C(+) circulating monocyte-depleted mice was accompanied with lower expression of CCR2 in CD11b(+) myeloid cells (Fig. 9d, right panel) and CXCL15 in BAL and lungs (Fig. 9e) (*p* < 0.05 v.s. the Group), further indicating the important function of monocyte chemotaxis in the progression of ALI.

## Discussion

In this study, we studied the role of SOCS3 in Ly6C(+) macrophage subtype differentiation in LPS-induced ALI mouse model. Our results revealed that Ly6C(+) macrophage subtypes were significantly increased in the ALI mouse model and the population was further increased in the mouse model that lacks the expression of SOCS3 in myeloid cell lineage. The increased Ly6C(+) macrophages were associated with more infiltration of Ly6G(+) neutrophils and Thy1.2 lymphocytes into the inflamed lung tissues. Accordingly, production of pro-inflammatory cytokines and mediators, such as IL-6, CXCL15 and IL-1beta was also elevated. Thereby, lack of SOCS3 expression in macrophages enhanced disease severity of ALI. The detrimental effects may be mediated by increasing pulmonary population of inflammatory Ly6C(+) cells, by which more neutrophils and lymphocytes were recruited into the inflamed lung tissues. In addition, we found more populations of neutrophils and lymphocytes in the naïve mice lack of SOCS3 protein expression. The results indicated the important roles of constitutive expression of SOCS3 and downstream signaling pathways in controlling lung inflammatory responses under physiological condition. It should be noted that the results were obtained in LPS-induced ALI mouse model, that may be not generalizable to other types of acute lung injury, for example oxygen free radical injury, etc. Previous studies showed that Ly6C(+) cells are derived from chemokine receptor CCR2-dependent macrophages of bone marrow [18]. After activation by pathogen exposure, the cells can be recruited into inflamed tissues and express many pro-inflammatory cytokines, such as TNF-alpha and other cytokines, responsible for tissue damage [25, 26]. In contrast, Ly6C(−) macrophages are resident macrophage-like cells that can proliferate and exert Nr4a1 (Nur77) transcription factor and IL-10-dependent anti-inflammatory function [15, 27]. According to the previous reports, Ly6C(+) and Ly6C(−) macrophage subtypes are reversible and share similar properties with M1 and M2 macrophages [28, 29]. Our experiments in vitro and in vivo also indicated that anti-inflammatory CD206(+) M2 macrophages were moderately reduced and pro-inflammatory M1 macrophages were moderately increased in ALI mouse model. M1 macrophage subtypes were changed similarly as Ly6C(+) macrophage subtypes within mice with ALI. The role of Ly6C(+) macrophages was also previously reported in a mouse model with spinal cord injury and amyotrophic lateral sclerosis (ALS), by which Ly6C(+) inflammatory macrophages had M1 cell biomarkers and contributed to axon loss through expressing high levels of chemokine receptor CCR2 and variable cytokines [17].

In addition, we investigated whether SOCS3 affected Ly6C(+) inflammatory macrophage differentiation and migration. To address this issue, we created SOCS3(Lyz2cre) KO mice with myeloid cell-restricted SOCS3 deficiency. The Ly6C(+) macrophage population was analyzed by flow cytometry analysis in the LPS-induced ALI mouse model. The results revealed that lack of SOCS3 expression significantly increased Ly6C(+) macrophage population in the SOCS3 KO mice 2 days after LPS i.t. treatment. The increased Ly6C(+) phenotypes were associated with more severe acute lung injury and pulmonary neutrophil infiltration. This suggested that SOCS3 may negatively control Ly6C(+) cell differentiation and/or migration. Our additional study by adoptive cell transfer further indicated that more monocyte migration from blood circulation into the lung tissues contributed to the increased population of pulmonary Ly6C(+) macrophages in the LPS-induced mice with ALI. The increased migration was supported by more expression of CCR2, CD80, CXCL15 and MCP-1 on the activated macrophages. The results displayed an important role of SOCS3 in regulation of macrophage chemotaxis in vivo. It should be noted that Lyz2cre only resulted in 40% (one cre) or 80% (two cre) loss of any given fl/fl gene in monocytes. Therefore, the effects by monocytes are only at best due to 80% loss of SOCS3. Secondary, Lyz2cre also resulted in SOC3 loss in neutrophils, dendritic cells and type II epithelial cells to lesser extent (data not shown). Therefore, more population of Ly6C(+) macrophages in SOCS3(Lyz2cre) mice may be not simply caused by SOC3 deficiency in macrophages alone. Other myeloid cell types may be involved in the process. The increased Ly6C(+) monocyte migration from blood circulation may contribute to a higher population of pathogenic Ly6C(+) macrophage in the inflamed lung of KO mice with ALI. However, we cannot exclude that Ly6C(+) cell in situ proliferation and polarization from Ly6C(−) cells may contribute to the increased Ly6C(+) cell population. Therefore, more investigation should be performed to clarify the issue in the future.

Butovsky et al. previously reported that depletion of Ly6C(+) cells by anti-Ly6C monoclonal antibody attenuated the severity of spinal cord injury in an amyotrophic lateral sclerosis (ALS) mouse model. The beneficial effects were associated with lower recruitment of Ly6C(+) monocyte into the injured spinal cord. The results indicated the pathological property of Ly6C(+) macrophages in the animal model [17]. Similar results were also confirmed in our ALI mouse model, in which ALI severity was reduced at least 2-fold, accompanied with lower neutrophil infiltrates and CXCL15 production after depletion of circulating Ly6C(+) monocyte in the LPS-treated SOCS3 KO mice. Thus, Ly6C(+) monocytes may be presented as potential therapeutic target cells in the treatment of ALI/ARDS by modulation of the cell type population and migration. It should be noted that Ly6C is expressed on other cell types such as granulocytes and dendritic cell precursors, a wide range of endothelial cells, subpopulations of B and T lymphocytes [30–32]. Thus, other Ly6C(+) cell types except for Ly6C(+) monocytes may be depleted in mice. Thus we conclude that Ly6C(+) cells, at least Ly6C(+) macrophages, are pro-inflammatory in LPS-induced ALI. Lack of SOCS3 expression enhanced ALI possibly by increasing proliferation and migration of pro-inflammatory Ly6C(+) subtype macrophages.

However, the underlying molecular mechanisms of how SOCS3 deficiency increased Ly6C(+) macrophages are not well defined so far. SOCS3 is an intracellular transcription factor that is positively regulated by IL-6 and IL-10 [9]. IL-6 increases SOCS3 expression through activation of JAK/STAT3 signaling pathways [9, 33]. We and other groups observed the increased SOCS3 expression and activation in ALI mouse model [7, 11], that should be mediated by IL-6 signaling pathway. As a negative regulator of JAK/STAT3 signaling pathway, the increased SOCS3 expression in ALI has an important implication in avoiding excessive inflammation and tissue damage. Thus, an optimal balance between SOCS3 expression level and JAK/STAT3 signaling pathway is critical for inflammation control at the later phase of ALI. We speculate that the unbalanced SOCS3 expression and JAK/STAT3 signaling may contribute to the uncontrollable lung inflammation in critically ill ARDS patients. Further analysis of SOCS3 expression level in human subjects would help dissect the underlying molecular mechanisms of uncontrollable lung inflammation in ARDS patients. Over-expressing SOCS3 or up-regulation of SOCS3 expression by molecular intervention would be considered a promising therapeutic approach in suppressing the uncontrollable lung inflammation among patients with ARDS.

Taken together, we conclude that myeloid cell-restricted lack of SOCS3 induced activation of JAK/STAT3 signaling, Ly6C(+) macrophage differentiation and migration, ultimately improving LPS-induced ALI. Over-expression of SOCS3 protein in macrophages may be considered a useful approach in suppressing ALI and the uncontrolled lung inflammation.

## Conclusion

In conclusion, myeloid cell-restricted lack of SOCS3 induced more severe ALI, in association with a higher population of Ly6C(+) subtype macrophages and enhanced STAT3 signaling pathway. Adoptive transfer of SOCS3-deficient BMDMs exaggerated ALI in recipient mice, whereas depletion of Ly6C(+) monocytes attenuated the severity of ALI. The results provide insight into a new role of SOCS3 in modulation of Ly6C(+) monocyte phenotypes in a mouse model with ALI, and offer a rationale for ALI immunotherapy by molecular intervention of macrophage subtypes.

## References

[1] MacLaren R, Jung R (2002). Stress-dose corticosteroid therapy for sepsis and acute lung injury or acute respiratory distress syndrome in critically ill adults. Pharmacotherapy.

[2] Qiu Z, Hu J, Van den Steen PE, Opdenakker G (2012). Targeting matrix metalloproteinases in acute inflammatory shock syndromes. Comb Chem High Throughput Screen.

[3] Fujino N, Kubo H, Suzuki T, He M, Suzuki T, Yamada M, Takahashi T, Ota C, Yamaya M (2012). Administration of a specific inhibitor of neutrophil elastase attenuates pulmonary fibrosis after acute lung injury in mice. Exp Lung Res.

[4] Puig F, Herrero R, Guillamat-Prats R, Gomez MN, Tijero J, Chimenti L, Stelmakh O, Blanch L, Serrano-Mollar A, Matthay MA, Artigas A (2016). A new experimental model of acid- and endotoxin-induced acute lung injury in rats. Am J Physiol Lung Cell Mol Physiol.

[5] Kobayashi K, Horikami D, Omori K, Nakamura T, Yamazaki A, Maeda S, Murata T (2016). Thromboxane A2 exacerbates acute lung injury via promoting edema formation. Sci Rep.

[6] Zhou GJ, Zhang H, Zhi SD, Jiang GP, Wang J, Zhang M, Gan JX, SW X, Jiang GY (2007). Protective effect of raloxifene on lipopolysaccharide and acid- induced acute lung injury in rats. Acta Pharmacol Sin.

[7] Jiang Z, Zhou Q, Gu C, Li D, Zhu L. Depletion of circulating monocytes suppresses IL-17 and HMGB1 expression in mice with LPS-induced acute lung injury. Am J Physiol Lung Cell Mol Physiol. 2017;312(2): L231–L242. 10.1152/ajplung.00389.2016 27913426

[8] Chaves de Souza JA, Nogueira AV, Chaves de Souza PP, Kim YJ, Silva Lobo C, Pimentellopes de Oliveira GJ, Cirelli JA, Garlet GP, Rossa C (2013). SOCS3 expression correlates with severity of inflammation, expression of proinflammatory cytokines, and activation of STAT3 and p38 MAPK in LPS-induced inflammation in vivo. Mediat Inflamm.

[9] Niemand C, Nimmesgern A, Haan S, Fischer P, Schaper F, Rossaint R, Heinrich PC, Muller-Newen G (2003). Activation of STAT3 by IL-6 and IL-10 in primary human macrophages is differentially modulated by suppressor of cytokine signaling 3. J Immunol.

[10] Qin H, Holdbrooks AT, Liu Y, Reynolds SL, Yanagisawa LL, Benveniste EN (2012). SOCS3 deficiency promotes M1 macrophage polarization and inflammation. J Immunol.

[11] Yan C, Ward PA, Wang X, Gao H (2013). Myeloid depletion of SOCS3 enhances LPS-induced acute lung injury through CCAAT/enhancer binding protein delta pathway. FASEB J.

[12] Speth JM, Bourdonnay E, Penke LR, Mancuso P, Moore BB, Weinberg JB, Peters-Golden M (2016). Alveolar epithelial cell-derived prostaglandin E2 serves as a request signal for macrophage secretion of suppressor of cytokine signaling 3 during innate inflammation. J Immunol.

[13] Zhao J, Yu H, Liu Y, Gibson SA, Yan Z, Xu X, Gaggar A, Li PK, Li C, Wei S (2016). Protective effect of suppressing STAT3 activity in LPS-induced acute lung injury. Am J Physiol Lung Cell Mol Physiol.

[14] Gordon P, Okai B, Hoare JI, Erwig LP, Wilson HM. SOCS3 is a modulator of human macrophage phagocytosis. J Leukoc Biol. 2016; 10.1189/jlb.3A1215-554RR 27106674

[15] Peng X, Zhang J, Xiao Z, Dong Y, Du J (2015). CX3CL1-CX3CR1 interaction increases the population of Ly6C(−)CX3CR1(hi) macrophages contributing to unilateral ureteral obstruction-induced fibrosis. J Immunol.

[16] Sunil VR, Francis M, Vayas KN, Cervelli JA, Choi H, Laskin JD, Laskin DL (2015). Regulation of ozone-induced lung inflammation and injury by the beta-galactoside-binding lectin galectin-3. Toxicol Appl Pharmacol.

[17] Butovsky O, Siddiqui S, Gabriely G, Lanser AJ, Dake B, Murugaiyan G, Doykan CE, PM W, Gali RR, Iyer LK (2012). Modulating inflammatory monocytes with a unique microRNA gene signature ameliorates murine ALS. J Clin Invest.

[18] Hettinger J, Richards DM, Hansson J, Barra MM, Joschko AC, Krijgsveld J, Feuerer M (2013). Origin of monocytes and macrophages in a committed progenitor. Nat Immunol.

[19] Schiwon M, Weisheit C, Franken L, Gutweiler S, Dixit A, Meyer-Schwesinger C, Pohl JM, Maurice NJ, Thiebes S, Lorenz K (2014). Crosstalk between sentinel and helper macrophages permits neutrophil migration into infected uroepithelium. Cell.

[20] Zaslona Z, Przybranowski S, Wilke C, van Rooijen N, Teitz-Tennenbaum S, Osterholzer JJ, Wilkinson JE, Moore BB, Peters-Golden M (2014). Resident alveolar macrophages suppress, whereas recruited monocytes promote, allergic lung inflammation in murine models of asthma. J Immunol.

[21] Amsellem V, Abid S, Poupel L, Parpaleix A, Rodero M, Gary-Bobo G, Latiri M, Dubois-Rande JL, Lipskaia L, Combadiere C, Adnot S (2017). Roles for the CX3CL1/CX3CR1 and CCL2/CCR2 chemokine Systems in Hypoxic Pulmonary Hypertension. Am J Respir Cell Mol Biol.

[22] Engel DR, Krause TA, Snelgrove SL, Thiebes S, Hickey MJ, Boor P, Kitching AR, Kurts C (2015). CX3CR1 reduces kidney fibrosis by inhibiting local proliferation of profibrotic macrophages. J Immunol.

[23] Satoh T, Nakagawa K, Sugihara F, Kuwahara R, Ashihara M, Yamane F, Minowa Y, Fukushima K, Ebina I, Yoshioka Y (2017). Identification of an atypical monocyte and committed progenitor involved in fibrosis. Nature.

[24] Jiang Z, Zhu L (2016). Update on the role of alternatively activated macrophages in asthma. J Asthma Allergy.

[25] Bain CC, Bravo-Blas A, Scott CL, Gomez Perdiguero E, Geissmann F, Henri S, Malissen B, Osborne LC, Artis D, Mowat AM (2014). Constant replenishment from circulating monocytes maintains the macrophage pool in the intestine of adult mice. Nat Immunol.

[26] Borthwick LA, Barron L, Hart KM, Vannella KM, Thompson RW, Oland S, Cheever A, Sciurba J, Ramalingam TR, Fisher AJ, Wynn TA (2016). Macrophages are critical to the maintenance of IL-13-dependent lung inflammation and fibrosis. Mucosal Immunol.

[27] Morias Y, Abels C, Laoui D, Van Overmeire E, Guilliams M, Schouppe E, Tacke F, deVries CJ, De Baetselier P, Beschin A (2015). Ly6C- monocytes regulate parasite-induced liver inflammation by inducing the differentiation of pathogenic Ly6C+ monocytes into macrophages. PLoS Pathog.

[28] Girgis NM, Gundra UM, Ward LN, Cabrera M, Frevert U, Loke P (2014). Ly6C(high) monocytes become alternatively activated macrophages in schistosome granulomas with help from CD4+ cells. PLoS Pathog.

[29] Graubardt N, Vugman M, Mouhadeb O, Caliari G, Pasmanik-Chor M, Reuveni D, Zigmond E, Brazowski E, David E, Chappell-Maor L (2017). Ly6Chi monocytes and their macrophage descendants regulate neutrophil function and clearance in acetaminophen-induced liver injury. Front Immunol.

[30] Geissmann F, Manz MG, Jung S, Sieweke MH, Merad M, Ley K (2010). Development of monocytes, macrophages, and dendritic cells. Science.

[31] Loomans CJ, van Haperen R, Duijs JM, Verseyden C, de Crom R, Leenen PJ, Drexhage HA, de Boer HC, de Koning EJ, Rabelink TJ (2009). Differentiation of bone marrow-derived endothelial progenitor cells is shifted into a proinflammatory phenotype by hyperglycemia. Mol Med.

[32] Marshall HD, Chandele A, Jung YW, Meng H, Poholek AC, Parish IA, Rutishauser R, Cui W, Kleinstein SH, Craft J, Kaech SM (2011). Differential expression of Ly6C and T-bet distinguish effector and memory Th1 CD4(+) cell properties during viral infection. Immunity.

[33] Boontanrart M, Hall SD, Spanier JA, Hayes CE, Olson JK (2016). Vitamin D3 alters microglia immune activation by an IL-10 dependent SOCS3 mechanism. J Neuroimmunol.

